# Modified Short Proximal Femoral Nail for Intertrochanteric Fractures of Femur in Indian Patients - our Experience

**DOI:** 10.5704/MOJ.2007.015

**Published:** 2020-07

**Authors:** V Jha, T Ahmed

**Affiliations:** 1 Department of Orthopaedics, Maharishi Markandeshwar Medical College and Hospital, Solan, India; 2 Department of Orthopaedics, Apollo Gleneagles Hospital, Kolkata, India

**Keywords:** proximal femoral nail, tip apex distance, lag screw position, lateral slide of lag screw, Asian

## Abstract

**Introduction:**

Proximal femoral nail (PFN) is a commonly used implant for intertrochanteric fractures which is designed according to western femoral measurements. However, anthropometry of proximal femur in Indian and in general, Asian, are smaller. So a modified short PFN with smaller dimensions was developed. This study analyses the radiological and functional outcome of treatment of intertrochanteric fractures with modified short PFN.

**Materials and Methods:**

A retrospective study analysed 120 adult patients operated between 2014-2017 using modified short PFN for intertrochanteric fractures, having a minimum follow-up of 12 months. Clinical and radiological parameters including tip-apex distance (TAD), position of tip of lag screw in femoral head, lateral slide of lag screw as well as length of anti-rotation screw were measured. Final functional outcome was assessed using Barthel’s index and Kyle’s criteria.

**Results:**

Good reduction was achieved in 90.83% cases and 79.16% had ideal placement of lag screw in femoral head. Intra-operative difficulties were encountered in 13.33% (n=16). Mean TAD AP (anteroposterior) was 11.8mm, TAD LAT (lateral) was 11.0mm and mean TAD TOT was 22.8mm. Overall mean lateral slide was 3.20mm and it was more in unstable fracture. We had five mechanical failures, one patient with screw breakage without loss of reduction and two peri-implant fractures after union. 81.66% returned to pre-injury levels of activity with 88.33% good to excellent outcome as per Kyle’s criteria.

**Conclusion:**

Although, not devoid of complications, modified short PFN results in good functional recovery of patients with intertrochanteric fractures of femur.

## Introduction

Intertrochanteric fracture (IT) of femur is a very commonly encountered orthopaedic condition especially in geriatric population^1^. Conservative management of these fractures are fraught with complications of prolonged recumbency as well as limp and shortening due to malunion in coxa-vara^2^. Extramedullary implants such as DHS, once considered the solution to these fractures have performed less than satisfactorily in unstable patterns paving way for intramedullary implants^3-5^. One such intramedullary implant currently in vogue is proximal femoral nail (PFN). Proximal femoral nail (Synthes) was designed keeping in mind the western population and comes in size of 240mm length, 17mm proximal diameter, distal diameter of 10-12mm and sizes of proximal and distal cephalic screws being 11mm and 6.5mm respectively. Study by Su *et al* demonstrated marked variability in location of femoral isthmus across various ethnic groups^6^. Siwach , in his study on 150 femoral bones, demonstrated smaller measurements related to proximal femoral and isthmus in Indian femurs. He recommended, in order to reduce incidence of intra-operative complications like fractures and splintering, implants need modifications according to Indian anthropometry. He recommended cephalomedullary nail to be adapted to dimensions described by Leung *et al*^7^. Leung *et al* used modified gamma nail for use in east Asian population and demonstrated improved clinical results in their multicentric study^8^. Indian femurs are proven to have considerably smaller anthropometric measurements compared to western population, thus requiring a smaller implant^9^. Pathrot and colleagues advised certain modifications in the short proximal femoral nail available in the Indian market^10^. Modified short proximal femoral nail also called trochanteric fixation nail (TFN) was introduced by Yogeshwar implants private limited for the purpose of Indian population and it works on the principles of PFN. Very few studies are published using this implant although it is being used very frequently^11, 12^.

We undertook this study to assess the clinical and radiological outcomes of intertrochanteric fractures of femur treated with modified short PFN.

## Materials and Methods

A retrospective study was conducted in patients that were operated at our institutes with modified short proximal femoral nail for IT fractures of femur between January 2014 to December 2017. Inclusion criteria for the study was kept as skeletally mature patient, with fresh (< 2 weeks) intertrochanteric fractures, treated operatively using modified short PFN, having a minimal follow-up of at least 12 months. Exclusion criteria were, fractures extending well below lesser trochanter, associated with other fractures and inadequate medical follow-up records or radiographs.

**Fig. 1:**
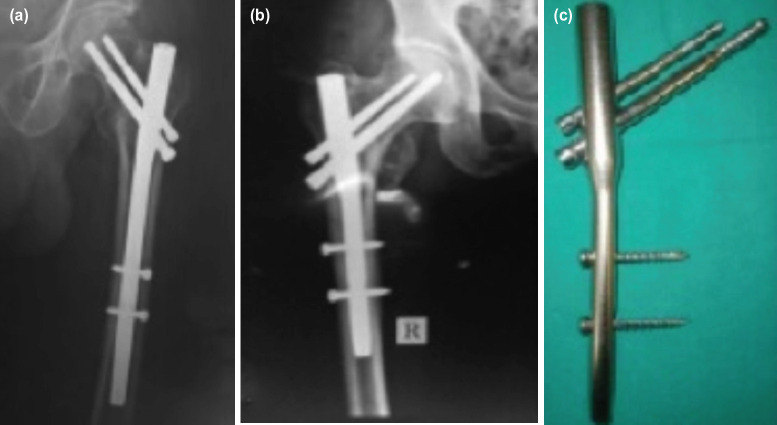
(a) radiograph of standard PFN, (b) radiograph of modified short PFN, (c) modified short PFN : compare how the standard PFN (250mm-on the left) crosses isthmus while modified short PFN stays well short of it.

A thorough search of records were done at the respective institutes of authors (both the institutes are tertiary care referral centres with dedicated trauma centres) and we found that 147 patients were operated with modified short PFN during this period. However 27 records were disqualified mainly due to inadequacies in radiographs, and lack of minimum 12 months follow-up. 120 records qualified for the study. The implant used was similar to PFN (Synthes), manufactured and distributed by Yogeshwar implants private limited (Thane), except it being smaller in size (Fig. 1). The implant is approved for use by Indian FDA. Nails were made of 316L stainless steel. Length of the nail used was 180mm with a proximal diameter of 15mm. Distal diameter had options of 9, 10 and 11mm. Two cephalic screws placed using jig measured 8.0mm (lag screw/lower screw/hip screw) and 6.4mm (anti-rotation screw/hip pin). Both dynamic and static options for 4.9mm bolts were present in distal locking and the jig allowed placement of distal bolts through the jig itself. Nails were designed with option of 130 degrees and 135 degrees neck shaft angle. The dimensions of this modified short PFN is smaller than standard PFN that comes in length of 240mm, proximal diameter of 17mm, distal diameter 10-13mm and cephalic screws measuring 11mm and 6.5mm (Fig 1).

Immediate post-operative radiographs were considered as baseline for subsequent implant related measurements. Tip apex distance (TAD), quality of reduction, position of tip of lag screws in head was done on immediate post-operative radiograph. Sequential follow-up radiographs were evaluated to assess union, position of screws and to calculate the lateral slide of lag screws. Magnification of the radiographs were calculated dividing true lag screw width by screw width measured on radiograph. All lengths measured were multiplied by this factor to account for magnification.

**Fig. 2:**
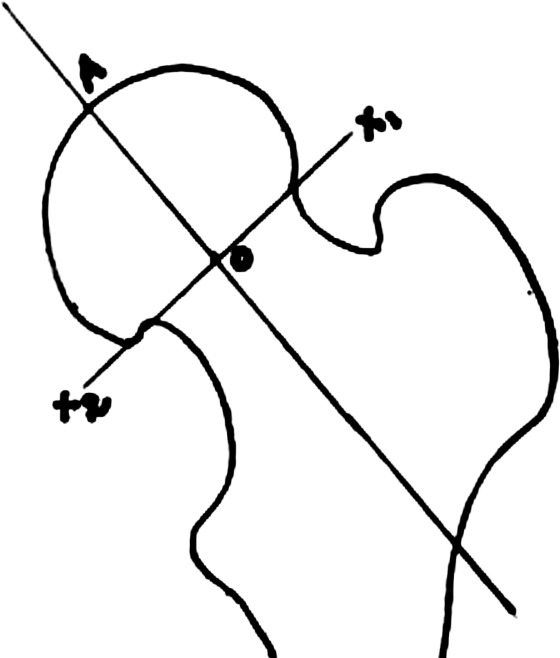
Defining the apex of femur head.

TAD was calculated by the method described by Baumgartner *et al*^13^ and adapted to cephalomedullary nail as described by Herman et al (Fig. 2)^14^. Apex was marked in both the views for calculation of TAD. The distance between the tip of screw and apex in that particular view was defined as TAD in that view. [TAD_total_ = TAD_AP_ + TAD_lateral_.] Baumgartner’s original description of TAD pertains with sliding hip screw system with a large single cephalic screw. It has been extrapolated and used in cephalomedullary nails including dual screw systems such as ours^15, 16^. TAD was measured for only the lag screw as hip pin gets obscured by the lag screw in lateral view. Baumgartner criteria^17^ was used to assess quality of reduction. Position of the tip of lag screw in the femoral head was assessed using Cleveland zones^18^. For the measurement of lateral slide of lag screw, immediate post op and final AP radiographs were compared as described by Morihara *et al*^19^.

Kyle’s criteria^20^ was used for final functional assessment while Barthel’s index^21^ was used to assess level of independence in activities of daily living. IBM SPSS Statistics version 26 was used for data analysis. Apart from descriptive statistics, Wilcoxon signed-rank test and Mann-Whitney U tests were used for analysis of nonparametric data.

## Results:

Demographic details are enlisted in Table I. Average age of the patients in our study was 71.45 years (range 30-95 years) with a median of 69.47 years. 56.66% were males while rest were females. Left and right side were affected in 40.83% and 59.17% respectively. Majority of fractures were of A2 type (68.3%). Table II enlists intra-operative details and findings. All patients were operated on a traction table and closed reduction was attempted. Only after failure of closed means (including joystick method), open reduction was done. Closed reduction was achieved in 98 patients, 14 needed joystick manoeuvre while 8 patients needed limited open reduction. Complete exposure of the fracture was not needed in any case. Predominantly 135° nail was used with 11mm diameter. There was a mean difference of 14.85mm in the sizes of the two cephalic screws used. The quality of reduction as per Baumgartner’s criteria on immediate post-operation radiographs was Good in 90.83% and no patient was classified as poor reduction. Toe-touch weight bearing with walker support was immediately started post-operatively and full weight bearing was undertaken only after radiological union.

**Table I:** Demographic details

Variables	Values	
Age (years)	Mean age 71.45 years (range 30-95 years)	
Sex	Male	56.66% (n=68)
	Female	43.33% (n=52)
Side affected	Left	40.83% (n=49)
	Right	59.16% (n=71)
Mode of injury	Trivial fall	95.83% (n=115)
	Road traffic accident	4.16% (n=5)
Pre-injury walking ability	Independent	88.33% (n=106)
	With support	11.66% (n=14)
Type of fracture AO/OTA	A1	27.5% (n=33)
	A2	68.3% (n=82)
	A3	4.16% (n=5)
Pre-anaesthesia ASA grading	A1+A2	43.33% (n=52)
	A3+A4	56.66% (n=68)
Duration of hospital stay	Mean 13.55 days	

**Table II:** Intra operative details

Variable	Value
Mean Duration of Surgery (min)	68.7 min (range: 32-140 minutes)
Mean Blood Loss (ml)	130ml (range: 50-350ml)
Reduction Method	Closed reduction in 98 patients
	14 patients underwent joystick manoeuvre 8 patients needed limited open reduction
Nail Angle Used	135° nail – 73.33 % ( n=88)
	130° nail - 26.66% (n=32)
Nail Diameter Used	Size 10mm - 21.66% (n=26)
	Size 11mm - 52.5% (n=63)
	Size 12mm - 25.83% (n=31)
Size Of 8.0mm(Lag) Screw	Mean: 95.10mm (80-110mm)
Size Of 6.4mm(Anti-rotation) Screw	Mean: 80.25mm (65-95mm)
Difference Between Lag Screw and Anti-rotation Screw	Mean: 14.85mm (5-25mm)
	10-15mm shorter anti-rotation screw were used in 87.5 %( n=105) cases.
Quality of Reduction	GOOD: 90.83% (n=109)
	ACCCEPTABLE: 9.16% (n=11)
	POOR: NONE

**Fig. 3:**
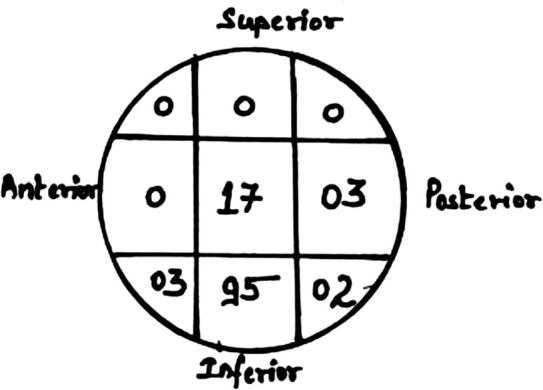
Position of lag screw in femoral head (chart).

We encountered certain procedure specific intra-operative difficulties in 16 cases and have been compiled in Table III. Most frequent was difficulty in inserting 6.4mm screw which was observed in 5% of cases. Mean TAD_total_ was 22.8mm while TAD_ap_ and TAD_lateral_ was 11.8 and 11.0mm respectively (Table IV). TAD has been poorly studied in biaxial cephalomedullary implants (having two screws) such as ours and currently there is no proven or recommended TAD for such implants^15, 16^. Most frequent position of lag screw was charted in inferior-central zone in 95 cases (Fig. 3). The next most commonly plotted position was central-central position and it was noted in 17 cases (14.16%).

**Table III:** Intra-operative complications

	Present series	Fogagnolo^23^	Tyllianakis^24^	Schipper^25^
	(n=120)	(n=46)	(n=45)	(n=211)
Difficulty inserting 6.4mm screw	6 (5%)	0	3(6.66%)	4(1.8%)
Fracture shaft of femur	0	0	1(2.22%)	0
Fracture Greater trochanter	0	4(8.6%)	1(2.22%)	0
Guide wire breakage	2 (1.67%)			
	8 bent*	2(4.3%)	0	0
	(6.67%)			
Difficulty inserting nail	0	2(4.3%)	1(2.22%)	0
Conversion to open Reduction	8 (6.67%)	1(2.2%)	3(6.66%)	17(8.1%)
Difficulty in distal locking	0	5(10.8%)	5(11.11%)	3(1.4%)

* 8 cases guide wires noticed to be bending while reaming and were removed before it could break

**Table IV:** Tip apex distance (post-op)

	Mean TAD_total_	Mean TADap	Mean TAD_lateral_
Present study (n=120)	22.8mm	11.8mm	11.0mm
Amir Herman *et al*^14^ (n=227)	20.3mm	9.7mm	10.0mm
Fogagnolo^23^ (n=46)	27.2mm	-	-
Metin Uzen *et al*^34^ (n=35)	24.2mm	-	-

Complications have been grouped and compiled in Table V. Reoperation was required in a total of 10 cases (8.3%). A total of five cases had screw cut-out and have been analysed in discussion part of the article with other cases of mechanical failure. One case was associated with deep infection. Peri-implant fracture was noted in two cases although it occurred after fracture consolidation. Isolated Z effect without loss of reduction was noted in four cases while most common complaint at final follow-up was thigh discomfort in 13 cases (10.8%). Average time to fracture union was estimated to be 17.32 weeks with average shortening noted to be 4mm. Nine patients (7.5%) had a shortening of one cm or more.

**Table V:** Complications

	No. of cases	Remarks
Deep infection + z effect + screw cut through	1	Occurred at 10 weeks. Implant removed and managed conservatively. Fracture united.
Z effect + screw cut through (no infection)	1	At eight weeks, implant removed and re-do with long PFN.
Screw Back out with loss of reduction	3	All occured within six weeks; one case re-do PFN was done while other two were managed with hemi-replacement arthroplasty
Periprosthetic Fracture	2	Both occurred after fracture consolidation and involved shaft- implant removal and IMIL nailing for shaft was done.
Screw breakage	1	Hip pin 6.0mm broken but fracture was consolidated without intervention
Superficial infection	2	Debridement and iv antibiotics resolved infection
Thigh discomfort after fracture union leading to implant removal	1	14 patients in total complained of thigh discomfort however only one was severe enough to merit implant removal (after union)
Reoperation	10	
Z effect without loss of reduction	4	
Isolated lateral thigh discomfort	13	

Mean pre-operative Barthel index was 98±4.501 while index at final follow-up was 91.37±13.349. Although, this is a statistically significant change (Wilcoxon signed-rank test, p=.000), clinically significant change in Barthel index was observed in 18.33% (22 cases). Ninety eight (81.66%) patients regained pre-injury status with minimal change in Barthel score (less than 5). Barthel index is scored on 10 parameters and assesses dependency of subjects in activities of daily living. It is scored on a 20-point scale and then result is multiplied by 5 to yield a score out of 100. Minimal clinically important difference (MCID) for Barthel index has been reported as 1.85 (on the 20-point Barthel index) for stroke patients^22^. On a 100-point scale this MCID will become 9.25. To the authors’ knowledge , MCID for Barthel index has not been calculated for musculoskeletal injuries, so we decided to use this value for our study i.e. those with change in score of less than 9.25 were to be regarded as not clinically discernible. Good to excellent functional recovery in accordance with Kyle’s criteria was noted in 88.33% (106) cases. Excellent outcome was noted in 69 and good outcome in 37 patients. Twelve patients had fair outcome while two had poor outcome.

The overall mean lateral slide of compression screws was estimated to be 3.20mm (range 0 to 13mm), after exclusion of cases with screw failure/cut-out. Unstable fracture patterns had more slide than stable ones. A1 fractures had a mean slide of 2.30mm (0-4 mm) while A2 type had 3.42mm (0-13mm).

## Discussion

We searched other series for intra-operative complications and compared them in Table III. Fogagnolo *et al*^23^ used Arbeitsgemeinschaft für Osteosynthesefragen (AO/ASIF) PFN (240mm) in a series of 46 patients and reported intra-operative difficulties in as many as 14 cases (30.4%) with fractured greater trochanter in 4(8.6%) and difficulty in nail insertion in 2 cases (4.3%). We did not encounter any greater trochanter fracture or difficulty in insertion of nail and we attribute it to smaller dimensions of the nail. Tyllianakis *et al*^24^ in a series of 45 patients had difficulty in 14 patients (31.1%) using AO/ASIF PFN. They reported fracture shaft of femur as well as fracture greater trochanter and difficult nail insertion in one case each. We did not encounter any such issue. Schipper *et al*^25^ in his large sample of 211 had difficulty during insertion of proximal screw in a mere 1.8% cases, compared to our 5% and Tyllianakis *et al*^24^ 6.6%. Tyllianakis^24^ and Schipper^25^ also reported similar rate of conversion to open reduction as ours i.e. around 6%. As evident from the Table III, we did not encounter any difficulty in distal locking as all lockings in our series were done via instrumentation jig. Operative difficulties were seen in 12% cases by Domingo *et al*^26^. These studies used standard and long PFN and not the modified PFN, the absence of fracture shaft of femur, greater trochanter and difficulty inserting the nail in our present series appear noteworthy.

While drilling over the guide wire, slight bending of guide wire can occur especially when it reaches near subchondral bone. It may lead to breakage of the guide wire as we saw in two of our cases and the intraosseous broken tips could not be removed. Fogagnolo *et al*^23^ also had 2 guide wire breakages. Detection of guide wire bending early is important so that it can be removed before it actually breaks. In eight cases, we were able to retrieve the wire before breaking. In such cases, free reaming beyond the bend under image intensifier may be needed after removal of guide wire. Reinsertion of a straight guide wire is necessary for screw insertion.

Sometimes when the lower screw is placed in central portion on AP view, the proximal de rotation screw goes too superiorly. This situation may be compounded if native neck shaft angle is less than the angle of the implant or if varus reduction is accepted. Even after acceptable reduction, proximal fragment may be pushed into varus while inserting the nail and that may lead to such situations. Hence, constant watch over reduction is very important. The entry point of nail is tip of greater trochanter as it has a 6° of valgus in design. Inadvertent lateralisation of entry point not only pushes the fracture into varus but also creates mismatch of neck shaft angle between nail and bone. This leads to derotation screw trajectory going much cranial and sometimes even perforating the neck.

Under reaming and excessive force application during nail insertion may cause fracture shaft of femur at the tip of the nail. This also occurs due to mismatch of femoral bow and nail when it crosses the isthmus. This is true when standard implants are used in Asian patients. Yaozeng *et al*^27^ reported that femoral shaft fractures were observed in 6 of the 107 patients with intertrochanteric fractures in their study. The nail used in current study did not cross the isthmus and no flexible reaming of medullary canal was needed. Only proximal reaming with cannulated hand held reamer sufficed the purpose. Lower dimensions of the nail averted this potentially disastrous complication.

**Fig. 4:**
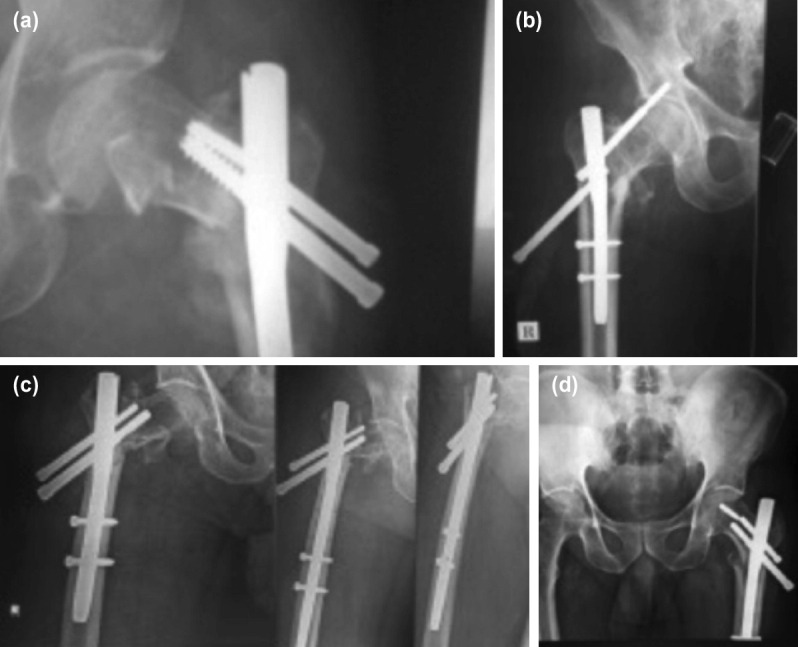
(a) Complication - loss of reduction with screw back out; no breakage. (b) Complication - ‘Z effect’. (c) Complication - screw back out in primary modified short PFN (left) as well as re-do with standard PFN in the same patient. (d) Complication - Screw cutout and breakage with loss of reduction.

Complete list of important complications encountered is tabulated in Table V. Fig. 4(a-d) demonstrates individual complications. Previous reports^23-30^ of secondary surgeries after PFN with varying frequencies ranging from 3.3% by Domingo *et al*^26^ to 28.8% by Tyllianakis^24^. Banan *et al*^28^ reported 6.5% resurgery rate while Schipper *et al*^25^ and Fogagnolo *et al*^23^ reported at 18.4% and 20% respectively. Our study reports reoperation rate of 8.3% .

Screw cut out incidences vary in literature. Tyllianakis *et al*^24^ had one failure due to screw cut-out out of 46 fractures while Simmermacher *et al*^29^ had one in 191 patients (both studies used AO/ASIF PFN). Domingo *et al*^26^ showed cut-out in 4/295 and Alyassari *et al*^30^ in 4/76 , whereas Schipper *et al*^25^ found 11 failures in 211 patients. Boldin *et al*^31^ studied a sample size of 55 patients and found 3 ‘z effect’, 2 ‘reverse z effect’ and 2 screw cut-outs ( attributed to smaller screw size in the neck. In spite of a sample size of 87 patients, Morihara *et al*^19^ did not report even a single cut-out of screws (not even Z effect) leading to conclusion that anti-rotation screws being 10-15mm shorter than the lag screw prevented the cut-out. Multiple predictors of screw cut-out has been described. Using multivariate logistic regression analysis Escolar *et al* found TAD, suboptimal osteosynthesis and distal static locking as predictive factors for screw cut-out^32^. Kashigar *et al* used univariate analysis and found TAD, calcar-TAD, Parker’s ratio index and neck angle difference to be associated with screw cut-outs in cephalomedullary nails^33^. John *et al* studied and included biaxial cephalomedullary nails in addition to uniaxial nails. They concluded that a combination of high TAD, suboptimal position of implant and poor restoration of neck shaft angle may predispose to cut-out. However, achieving TAD within safe limits didn’t appear to influence screw and device migration in dual screw nails^16^. Another technical aspect of note is the length of anti-rotation screw. PFN being a twin screw construct, the smaller screw (proximal hip pin, 6.4mm) serves the purpose of providing rotational stability while the lag screw serves load bearing function. When hip pin protrudes beyond lag screw, increased vertical forces induce Z-effect (aka Knife effect) forcing the proximal screw medially into the joint and distal lag screw to slide back laterally.

Screw cut-out rate was 5/120 in our series. One of which was associated with infection, and four without infection. We analysed TAD, position of lag screw as well as relative length of anti-rotation screw on post-operative radiographs in the screw cut-out cases. Overall TAD_total_ was found to be 22.8mm which was less than Fogagnolo *et al*^23^ and Uzen *et al*^34^ but more than Herman *et al*^14^ (Table IV).

Calculations of mean TAD (Table VI) reflected higher values in the group with screw cut-out when compared with the one without cut-out. Mann Whitney-U test suggested that TAD_lat_ and TAD_total_ were significantly different in the two groups, while TAD_ap_ showed a trend towards significance. Baumgartner^13^ recommended TAD to be less than 25mm, albeit in a single screw construct. Some authors^23, 34^ have found correlation of large TAD in PFN (a double screw construct) with screw cut-outs while others^14^ have refuted its use for PFN. Most of the authors however do concur with the fact that tip of lag screw must be as close to subchondral bone as possible. TAD represents both the position and depth of a screw in the femoral neck and head and was shown to be the most important predictive factor for the occurrence of a cut-out^35, 36^. Geller *et al*^37^ reported a high incidence (44%) of cut-outs in intertrochanteric fractures that were surgically fixed with a TAD of >25mm.

**Table VI:** Comparison of TAD in screw cut out group vs non cut out

	Without cut out of screws (n=115)	Cases with screw cut out (n=5)	Mann Whitney U test (p value)
Mean TADap	11.6 mm	14.4 mm	.093
Mean TADlat	10.9 mm	14.1 mm	.013
Mean TADtot	22.5 mm	28.5 mm	.021

Ideal placement of lag screw in head is suggested to be Inferior-Central^19^. Kyuzyk *et al* demonstrated that biomechanical stiffness is maximised when lag screw is placed inferiorly in AP view, and central placement in lateral view maximises its load to failure^38^. This position was observed in 79.16% (95/120). The next most commonly plotted position was central-central position and it was noted in 17 cases (14.16%).

Sub optimal position of screw in Cleveland quadrants may have a contributing effect in the screw cut-out. Zirngibl *et al*^39^ compared screw cut-out cases with controls and found increased odds risk with lag screw position in cranial, anterior and posterior thirds of the screw. However the results did not reach statistical significance. They advocated placement in the central third of the femoral head. In our study, out of four cases where screw cut-out had occurred without infection, in three cases, the tip of lag screw was in central-central quadrant and one in central posterior quadrant. Helwig *et al*^40^ advocated advantages of cranial position in his study and is in contradiction to our findings. Further studies are required in biaxial systems to determine optimal position of the screws. However inferior central zone appears to be the safest and therefore maintenance of appropriate neck shaft angle and position of lag screw in inferior quadrant is very important aspect of the technique. Both are intricately connected as angle of screw placement is inherent to the design of neck and that is prefixed, hence unless correct neck shaft angle is achieved, screw insertion may prove to be very tricky. This again emphasises on achieving as near anatomical reduction as possible.

As mentioned before, length of derotation screw has been reported as predictive factor for cut-out. Morihara *et al* recommended that derotation screw must be at least 10-15mm shorter than the larger lag screw^19^. Zirngibl *et al*^39^ analysed this by drawing an imaginary line from tip of lag screw to the tip of nail and proved that anti-rotation pin protruding beyond this line had a significantly high odds ratio of 8.8 for fixation failure. They go on to suggest that, this could be the most important factor influencing the screw cut-out or cut-through rates. Analysis of relative screw lengths in femoral head revealed that in four out of five cases of screw cut-out, the anti-rotation screw was advanced either beyond the tip of lag screw or was at the same level, thus leading to increased vertical forces on the anti-rotation pin.

In short, a combination of suboptimal position of lag screw in femoral head, high TAD as well as excessively long anti-rotation screw were found in cases that had fixation failures. The mean operative time found in this study was lesser than that reported by Fogagnolo (83.4 min) and Morihara (77min) who used standard PFN. Some studies do quote lesser operative time^41^, however, it is unclear what constitutes operative time in studies. Whether from incision to closure or from starting of attempt at closed reduction. In our study, we included the duration of closed reduction before incision as well. Mean blood loss is considerably lesser than that occurs with standard PFN^41-43^.

In authors’ opinion, in order to avoid screw cut-out and mechanical failure, effort needs to be directed at minimising TAD by inserting compression screw deep into the head up to 5mm below subchondral bone. In addition to ensuring adequate purchase in proximal fragment it also prevents inadvertently longer anti- rotation screws. Every effort must be directed towards careful placement of lag screw in ‘safe quadrant’ (inferior in AP and central in lateral view). Achieving appropriate anatomical reduction and not accepting even slight varus goes a long way in achieving this objective. Valgus reduction may be accepted, implant permitting, and may even be recommended in unstable fractures. In unstable fractures as union occurs, further impaction and varus occurs.

The overall mean lateral slide of compression screws was estimated to be 3.20mm (range 0 to 13mm), after exclusion of cases with screw failure/cut-out. This lateral slide was found out to be more in unstable fractures when compared to stable fracture patterns. A1 fractures had a mean slide of 2.30mm (0-4mm) while A2 type had 3.42mm (0-13mm). As union progresses, proximal fragment gets impacted onto distal fragment as well as the nail, leading to lateral slide of both the cephalic screws and can be a surrogate marker of collapse of the fracture. Any restriction in this lateral slide may initiate cut-out or joint penetration by the screws.

Despite these complications and mechanical failures, recovery to pre-injury functional status as per Barthel’s score was found in 81.66% of the cases (change less than MCID). As per Kyle’s criteria, good to excellent functional recovery was found in 88.33% (106) cases. Gadegon *et al*^11^ reported 90% excellent outcome while Pavelka *et al*^44^ had 92% excellent functional outcome.

In limitations, inherent to the methodology of the study which involves medical records examination, we could not use femur length as our inclusion criteria as these were not consistently mentioned in all the records. Another limitation of this study is a lack of control group.

## Conclusion

Modified Short Proximal Femoral nail needs careful preoperative plan, followed by expert intra-operative technique coupled with good reduction. If appropriately followed, it leads to high union rate with minimal soft tissue damage. Placement of screws needs special mention and are essential for successful outcome. Safe position of screw is inferior in AP plane and central in lateral view. TAD needs to be kept to minimum. Deep insertion of lag screw into femoral head, closer to subchondral bone with a shorter anti-rotation screw which doesn’t cross the tip of lag screw is equally important. Although, not devoid of complications, modified short PFN results in good functional recovery of patients with intertrochanteric fractures of femur. The shorter nail allows for easier insertion (no reaming required post isthmus) and lesser blood loss with lesser complication rates. Its shorter length renders it not suitable for fractures that extend far distal to lesser trochanter. Further studies are needed to compare the efficacy of shorter variant in Asians as well as compare it with new variant PFNA.
